# Supplementary material for: Design, and dynamic evaluation of a novel photovoltaic pumping system emulation with DS1104 hardware setup: Towards innovative in green energy systems
Source: PLoS One. 2024 Oct 7;19(10):e0308212. doi: 10.1371/journal.pone.0308212 (PMC11458027; doi:10.1371/journal.pone.0308212)
Supplement: Attachment — Submitted filename: REVIEW_PLOSEONE-solar water pump.pdf [file pone.0308212.s001.pdf]

Design, and Dynamic Evaluation of a Novel Photovoltaic Pumping System Emulation with DS1104 Hardware Setup: Towards Innovative in Green Energy Systems

This study introduces an emulation approach for photovoltaic (PV) Water Pumping(WP) systems. In this work integrates two emulators into a single chain, effectively combining PV and WP emulation while optimizing system dynamics. The goal to devise a comprehensive emulator for PV water pumping systems and assess an improved control algorithm. To regulate the water MP, we deploy an optimized scalar control strategy. Validation of this control strategy is conducted through an experimental arrangement utilizing the dSPACE control desk DS1104. Obtained results affirm the emulator's proficiency in faithfully reproducing genuine solar panel characteristics.

The following are comments/suggestions to improve the manuscript:

1. It seems the authors have some positive results. Please compare the newly adopted power point tracking algorithm(GSS) with the conventional one (perturb and observe) ; show the improvement on the power plots (on the same plots).

2. Nomenclature is should be written clearly. Some lines are miss-aligned or spelled wrongly.

3. Your figures are not in good quality/resolution(figure 4,7, and 9). Please use improved quality images for your simulation and experiments.

4. The literature review is not sufficient. Include the following papers on converter control, inverter and motor control as such as

   - Improved Model Predictive Speed Control of a PMSM via Laguerre Functions, Mathematical Problems in Engineering, vol. 2024, Article ID 5562771, 14 pages, 2024. https:// doi.org/ 10.1155/2024/5562771.
   - Stabilization and Voltage Regulation of the Buck DC-DC Converter Using Model Predictive of Laguerre Functions, Studies in Informatics and Control, ISSN 1220-1766, vol. 26(3), pp. 315-324, 2017. https://doi.org/10.24846/v26i3y201707
   - A Modified Controller for Three Level Three-Phase Voltage Source Inverter based on Laguerre Functions. International Journal of Computer Applications, 182(25), 21–28. https://doi.org/ 10.5120/ijca2018918081.

5. There are typos Typos.
   - In the abstract, The first one is a PV system emulator that employs ~~back~~ buck converter control to faithfully mirror the characteristics of PV panels.
6. Please write in passive speech. Avoid pronouns we, their, our, them etc.
7. Lastly not least, plot the simulation and experimental results on the same figure/plots so that your experiments can validate the simulation results.
